# Repetitive milrinone therapy in ambulatory advanced heart failure patients

**DOI:** 10.1002/clc.23802

**Published:** 2022-03-04

**Authors:** Michal Laufer‐Perl, Sapir Sadon, David Zahler, Assi Milwidsky, Ben Sadeh, Orly Sapir, Yoav Granot, Liuba Korotetski, Liora Ketchker, Maayan Rosh, Shmuel Banai, Ofer Havakuk

**Affiliations:** ^1^ Cardiology Division, Tel Aviv Sourasky Medical Center affiliated to Tel Aviv University Tel Aviv Israel

**Keywords:** advanced heart failure, inotropes, milrinone

## Abstract

**Background:**

Advanced heart failure (HF) patients usually poorly tolerate guideline‐directed HF medical therapy (GDMT) and suffer high rates of morbidity and mortality. The use of continuous inotropes in the outpatient settings is hampered by previous data showing excess morbidity. We aimed to assess the safety and efficacy of repetitive, intermittent, short‐term intravenous milrinone therapy in advanced HF patients with an intention to introduce and up‐titrate GDMT and improve functional class.

**Hypothesis:**

Repetitive, intermittent milrinone therapy may assist with the stabilization of advanced HF patients.

**Methods:**

Advanced HF patients treated with beta‐blockers and implanted with defibrillators were initiated with repetitive, intermittent short‐term intravenous milrinone therapy at our HF outpatient unit. Patients were prospectively followed with defibrillator interrogation, functional class assessment, B‐natriuretic peptide (BNP) levels, and echocardiography parameters.

**Results:**

The cohort included 24 patients with a mean 330 ± 240 days of milrinone therapy exposure. Mean age was 73 ± 6 years with male predominance (96%). Following milrinone therapy, median BNP levels decreased significantly (882 [286−3768] to 631 [278−1378] pg/ml, *p* = .017) with a significant reduction in the number of patients with New York Heart Association (NYHA) Class III and IV (*p* = .012, 0.013) and an increase in number of patients on GDMT. Importantly, the number of total sustained ventricular tachycardia events and HF hospitalizations did not change.

**Conclusions:**

In this small cohort of advanced HF, repetitive, intermittent, short‐term milrinone therapy was found to be safe and potentially efficacious.

## 1 INTRODUCTION

Advanced heart failure (HF) represents the extreme of HF and bears grave prognosis.^1^ These patients usually poorly tolerate guideline‐directed HF medical therapy (GDMT) and may need to undergo definitive treatments (i.e., left ventricular assist device implantation or heart transplantation). Many of them, however, are found unsuitable for such therapies due to severe comorbidities or inappropriate settings related to the patients and their surroundings. In this case, supportive care is applied with an expectation to maintain a reasonable quality of life and many times incorporates the use of inotropic therapy. The choice of inotropic agents, their mode of delivery, the adjunct therapy, and, importantly, the goal of treatment may differ significantly between HF centers.^2^, ^3^, ^4^, ^5^, ^6^ In this small cohort of ambulatory advanced HF patients, we evaluated for the first time, the use of repetitive, intermittent short‐term, intravenous (IV) milrinone therapy with an aim to relieve symptoms and introduce and up‐titrate GDMT therapy.

## 2 METHODS

### 2.1 Study population

We performed a prospective, single‐center observational study at our HF outpatient unit at the Tel‐Aviv Sourasky Medical Center, a tertiary care center in Tel‐Aviv, Israel. All patients were asked to sign an informed consent allowing anonymous prospective data collection, including demographic, clinical, laboratory, and imaging parameters. This study is a part of our HF prospective registry, following all HF patients evaluated at the HF clinics of our facility and was approved by the local ethics committee (Identifier: 0574‐16‐TLV).

### 2.2 Settings

Our advanced HF outpatient unit is based on routine visits at a time interval of 7−14 days during which the patients are seen and examined by a dedicated team of nurses and physicians experienced with HF treatment. Laboratory and imaging evaluation is done according to patient's status. Appropriate therapy (e.g., inotropes, diuretics, iron, pleural/peritoneal tap) is applied following clinical evaluation and tests results.

### 2.3 Patients

The current cohort included consecutive patients defined as advanced HF with reduced ejection fraction (HFrEF) according to the following criteria: (a) left ventricular ejection fraction (LVEF) ≤ 35%; (b) elevated brain natriuretic peptide (BNP) levels > 150 pg/ml; and (c) NYHA class IIIb−IV, despite maximally tolerated GDMT. All patients were treated with beta‐blockers and had implanted defibrillators before the initiation of IV milrinone therapy.

### 2.4 Follow‐up and milrinone protocol

The following surveillance is part of the comprehensive treatment given at our advanced HF outpatient unit:

1. During milrinone infusion, participants were closely monitored both with electrocardiogram monitors and vital signs.
2. Patients were treated with once‐weekly intermittent IV milrinone 0.25−0.5 mcg/kg/min for a 6‐h period.
3. During routine clinic visit, patients were treated with IV furosemide and IV Iron according to the patient's clinical status and tests results.

During IV milrinone therapy, we reevaluated functional class, BNP levels, renal function, echocardiography parameters, presence and dose of GDMT, number of HF hospitalizations, and occurrence of ventricular tachycardia (VT). In case a patient was interrupted from milrinone therapy, the evaluation was performed at the last milrinone therapy exposure. Our aim was to introduce and/or up‐titrate GDMT to maximally tolerated doses.

### 2.5 Statistical methods

Descriptive results are expressed as means ± standard deviation (SD) in cases of normally distributed continuous variables, median and interquartile (IQR) range were used for non‐normally distributed ones. Categorical variables are expressed as absolute numbers and percentages. Differences in continuous variables between “before” and “after” onset of milrinone therapy were evaluated with paired samples *t *test if normally distributed and Wilcoxon signed rank test if not normally distributed. *χ* ^2^ test (or Fischer exact test when appropriate) was used for comparison of categorical variables.

## 3 RESULTS

From July 2018 to May 2021, 27 patients were treated with routine intermittent IV milrinone at our HF outpatient unit. Three patients were excluded from the cohort due to baseline LVEF > 35%, leaving a total of 24 patients. The mean age was 73 ± 6 years with male predominance (96%). Ischemic cardiomyopathy was the baseline etiology in 21 (87%) patients and hypertension and diabetes were frequent (75% and 67%, respectively). All patients were implanted with a defibrillator; 11 patients (46%) had also a cardiac resynchronization therapy device (CRTD) (Table 1). The median time between CRTD implantation to the introduction of milrinon therapy was 1225 [IQR: 464−2842] days. The mean baseline systolic blood pressure was 117 [IQR: 102−133] mm Hg and creatinine levels were 1.9 ± 0.6 mg/dl. Baseline LVEF was 27 ± 6%, left ventricular diastolic diameter was 65 ± 9 mm, and systolic pulmonary artery pressure (SPAP) was 51 ± 17 mm Hg (Table 2).

**Table 1:** Baseline characteristics of all patients

	All (*n* = 24)
Age, years, mean (± SD)	73 (±6)
Gender, male, *n* (%)	23 (96)
Ischemic etiology, *n* (%)	20 (83)
BMI, median (IQR)	26 (23−29)
Hypertension, *n* (%)	18 (75)
Diabetes mellitus, *n *(%)	16 (67)
Hyperlipidemia, *n* (%)	19 (79)
Atrial fibrillation, *n* (%)	9 (38)
Past or present smoker, *n* (%)	10 (42)
Chronic kidney disease, *n* (%)	13 (54)
Ischemic stroke, *n* (%)	5 (21)
ICD, *n* (%)	24 (100)
CRTD, *n* (%)	13 (54)

Abbreviations: BMI, body mass index; CRTD, cardiac resynchronization therapy defibrillator; ICD, implantable cardiac defibrillator; IQR, interquartile range; SD, standard deviation.

**Table 2:** Clinical, laboratory, and echocardiographic parameters changes during milrinone therapy

Variable	Pre‐milrinone (*n* = 24)	Post‐milrinone (*n* = 24)	*p* value
Clinical parameters			
Heart rate (bpm), mean (± SD)	70 ± 15	72 ± 13	.67
Systolic BP (mm Hg), median (IQR)	117 (102−133)	114 (96−121)	**.03**
Diastolic BP (mm Hg), mean (± SD)	61 ± 15	60 ± 11	.65
O_2_Sat (%), median (IQR)	98.1 ± 2.1	99.7 ± 1.4	.06
Laboratory parameters			
Hemoglobin (g/dl), mean (± SD)	12.1 ± 1.8	12.3 ± 1.6	.61
White blood cells (10^3^/µl), median (IQR)	6.8 (5.7−8.2)	6.7 (5.4−7.4)	.18
Platelets (10^3^/µl), median (IQR)	162 (139−211)	159 (122−210)	.13
RDW (%), median (IQR)	16.0 (14.6−17.9)	15.3 (14.1−16.5)	**.04**
Creatinine (mg/dl), mean (± SD)	1.9 ± 0.6	2.1 ± 0.9	**.04**
BUN (mg/dl), mean (± SD)	46 ± 22	56 ± 31	**.06**
BNP (pg/ml), median (IQR) (*n* = 20**)**	882 (286−3768)	631 (278−1378)	**.017**
Sodium (mmol/L), median (IQR)	137 (137−139)	138 (134−139)	.41
Potassium (mmol/L), median (IQR)	4.4 (4.1−4.8)	4.5 (4.1−4.8)	.56
Iron (mcg/dl), mean (± SD)	51 ± 20	57 ± 23	.38
Transferrin (mg/dl), mean (± SD)	231 ± 43	203 ± 36	.01
Transferrin saturation (%), mean (± SD)	16 ± 6	19 ± 6	.09
Ferritin (ng/ml), median (IQR)	210 (139−516)	370 (264−513)	.27
Echocardiography			
LVEF (%), mean (± SD)	27 ± 6	27 ± 7	.88
LVESD (mm), mean (± SD) (*n* = 13)	54 ± 8	55 ± 8	.77
LVEDD (mm), mean (± SD) (*n* = 13)	65 ± 9	65 ± 7	.65
LV mass (g), mean (± SD) (*n* = 7)	316 ± 116	303 ± 90	.63
Deceleration time (ms), mean (± SD (*n* = 15)	193 ± 79	215 ± 91	.34
IVS (mm), median (IQR) (*n* = 16)	11 (8−12)	10 (9−12)	.37
SPAP (mm Hg), mean (± SD)	51 ± 17	49 ± 17	.37
LA volume (ml), mean (± SD) (*n* = 18)	113 ± 48	113 ± 46	.93
LA volume index (ml/m^2^), mean (± SD) (*n* = 17)	59 ± 29	61 ± 29	.38
e’ septal (cm/s), mean (± SD) (*n* = 13)	4.12 ± 1.15	4.27 ± 1.84	.69
e’ lateral (cm/s), median (IQR) (*n* = 13)	5.7 (5.0−7.0)	5.2 (3.6−7.6)	.93
E/e’ septal, median (IQR) (*n* = 13)	26 (16−31)	21 (15−29)	.08
E/e’ lateral, mean (± SD) (*n* = 13)	17 ± 6	15 ± 8	.29
E/e’ average, mean (± SD) (*n* = 14)	19 ± 6	17 ± 7	.15

Abbreviations: SD, standard; BNP, brain natriuretic peptide; BP = blood pressure; BUN, blood urea nitrogen; IQR, interquartile; IVS, intraventricular septum; LV, left ventricle; LVEDD, left ventricular end diastolic diameter; LVEF, left ventricle ejection fraction; LVESD, left ventricular end systolic diameter; RDW, red cell distribution width; SD, standard deviation; SPAP = systolic pulmonary artery pressure.

At baseline, all patients were treated with beta‐blockers. The majority of the patients [75%] were treated with bisoprolol, and the remaining were treated with either metoprolol succinate [12.5%] or carvedilol [12.5%]. Overall, the mean dose of bisoprolol equivalent was 5 mg daily. Thirteen patients were treated with angiotensin receptor neprylisin inhibitor (ARNI), and three patients with angiotensin‐converting‐enzyme inhibitor (ACEI) or angiotensin II receptor blockers (ARB). Thirteen patients were treated with mineralocorticoid receptor antagonists (MRA) and 21 patients with furosemide with a mean dose of 120 [IQR: 80−140] mg (Table 3).

**Table 3:** Change in guideline‐directed HF medical therapy and functional class during milrinone therapy

Variable	Pre‐milrinone (*n* = 24)	Post‐milrinone (*n* = 24)	*p* value
ACEI, *n* (%)	2 (8)	1 (4)	—
ARB, *n *(%)	1 (4)	0 (0)	—
ARNI, *n* (%)	13 (54)	19 (80)	.07
ARNI dose (mg), median (IQR), mean (± SD) (only patients on ARNI before milrinone) (*n* = 13)	200 (100−350)	250 (100−400)	**.01**
	230 ± 118	320 ± 103	
BBs, *n* (%)	24 (100)	24 (100)	—
Bisoprolol, *n* (%)	18 (75)	18 (75)	
Other, *n* (%)	6 (25)	6 (25)	—
MRAs, *n* (%)	14 (58)	16 (67)	.55
MRAs dose (mg), median (IQR), mean (± SD)	25 (12.5−37.5)	12.5 (12.5−25)	.32
	25 ± 13	23 ± 14	
Statins, *n* (%)	18 (75)	18 (75)	—
Ezetimibe, *n* (%)	3 (13)	4 (17)	.70
Furosemide, *n* (%)	21 (88)	23 (96)	.61
Furosemide dose (mg), median (IQR)	120 (80−140)	80 (60−120)	.38
	107 ± 52	98 ± 43	
Metolazone, *n* (%)	6 (25)	6 (25)	—
Digoxin, *n* (%)	3 (13)	5 (21)	.70
Antiarrhythmic, *n* (%)	7 (29)	6 (25)	.74
Hydralazine, *n* (%)	4 (17)	6 (25)	.48
Nitrates, *n* (%)	1 (4)	4 (17)	.35
SGLT2 inhibitors, *n* (%)	5 (21)	14 (58)	**.01**
NYHA 3, *n* (%)	17 (71)	13 (54)	**.027**
NYHA 4, *n* (%)	7 (29)	0 (0)	**.01**
Hospitalizations (per year)	1.17 ± 1.16	1.16 ± 1.7	.98

Abbreviations: ACEI, angiotensin‐converting‐enzyme inhibitor; ARBs, angiotensin II receptor blockers; ARNI, angiotensin receptor neprylisin inhibitor; BB, beta‐blockers; IQR, interquartile; MRA, mineralocorticoid receptor antagonists; SD, standard deviation; SGLT2, sodium glucose transporter type 2.

**Figure 1:**
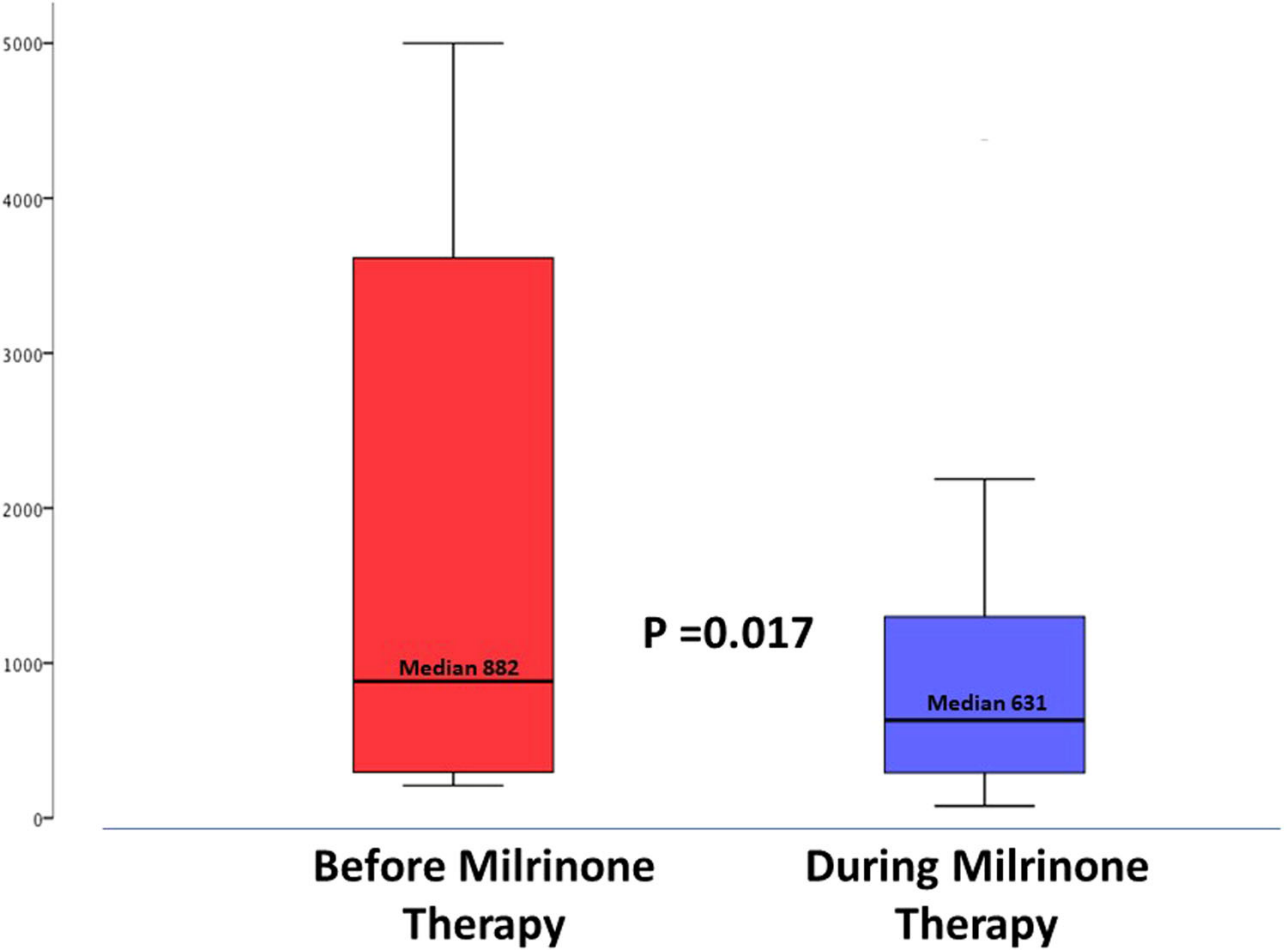
Following repetitive milrinone therapy, a significant reduction in brain natriuretic peptide levels was noted

The mean therapy time exposure to IV milrinone was 330 ± 240 days, with a median number of 25 [IQR: 20−44] therapies. Individual pre‐ and post‐milrinone therapy data are described in Tables 2 and 3. During milrinone therapy, the number of patients introduced to ARNI therapy increased from 13 to 19 (54% vs. 80%, *p* = .004) and the dose was increased from a median 200 [IQR: 100−350] to 250 [IQR: 100−400] mg, *p* = .01. As the cohort included advanced HF patients, the most common reasons for non‐adherence to optimal doses of GDMT were worsening of kidney function (*n* = 4) and symptomatic hypotension (*n* = 4). Nineteen patients received intermittent IV furosemide according to their clinical condition and 15 patients received at least one dose of IV iron. Along with the follow‐up, oral or IV furosemide doses were reduced in eights patients and maintained in the rest of the group. At baseline, five patients were treated with type 2 sodium‐glucose transporter (SGLT2) inhibitors and this therapy was introduced to another nine patients under milrinone therapy (21% vs. 58%, *p* = .01). During follow‐up, mean BNP levels decreased from 882 [IQR: 286−3768] to 631 [IQR: 278−1378] pg/ml, *p* = .017 (Figure 1). The mean creatinine increased form 1.9 ± 0 to 2.1 ± 0.9 mg/dl, *p* = .04. Additionally, while mean LVEF remained unchanged (27 ± 6% vs. 27 ± 7%, *p* = .88), we observed a nonsignificant reduction in E/e' and SPAP (Table 2).

During IV milrinone therapy, we observed an improvement in symptoms and functional class, with the number of patients classified as NYHAIII and IV reduced from 71% and 29% to 54% and 0%, *p* = .027 and .01, respectively (Table 3). The number of HF hospitalizations per year, pre‐ and post‐milrinone therapy, remained stable (1.17 ± 1.17 vs. 1.16 ± 1.7, *p* = .98). Notably, during the pre‐milrinone period, 16 patients had a total of 28 hospitalizations whereas in the post‐milrinone period, 11 patients had a total of 22 hospitalizations. Out of these 22 hospitalizations, 8 were in fact of one patient.

Though 6‐min walk test or cardio‐pulmonary exercise test (CPET) were not part of our scheduled follow‐up, CPET results in 13 of our patients demonstrated a significant increase in exercise duration (238 ± 51 to 264 ± 57 s, *p* = .04) and a trend toward improvement in workload (119 ± 28 to 129 ± 36 W, *p* = .11) and in ventilatory efficiency (37.6 ± 3.8 to 36.2 ± 3.9, *p* = .11) following milrinone therapy (Table 4).

**Table 4:** CPET findings before and after milrinone exposure

CPET results, *n* = 13	Pre‐milrionon	Following milrinon	*p* value
VO_2_ max (ml/min/kg), mean ± SD	11.2 ± 1.6	11.8 ± 2.7	.45
Workload (W), mean ± SD	119 ± 28	129 ± 36	.11
Time (s), mean ± SD	238 ± 51	264 ± 57	.04
O_2_ pulse (ml/min/kg), mean ± SD	8.3 ± 1.1	8.5 ± 1.1	.64
VE/VCO_2,_ mean ± SD	37.6 ± 3.8	36.2 ± 3.9	.11
VE/VCO_2_ > 35, * n* (%)	8 (62)	6 (46)	.43

Abbreviations: CPET, cardiopulmonary exercise test; SD, standard deviation; VE/VCO_2_, ratio of total ventilation to carbon dioxide production; VO_2_ max, maximal oxygen consumption.

Examining the treatment effect on arrhythmias, sustained ventricular tachycardia (VT) occurred in six versus four patients pre‐ and post‐milrinone therapy, respectively. Two patients (in both time periods) required an implantable cardioverter defibrillator (ICD) shock therapy.

Since the number of exposures to milrinone therapy differed among the study participants, we also examined the reduction in BNP levels and the improvement in NYHA class between patients with “long” (>50% percentile) versus “short” (<50% percentile) therapy duration and found no statistically significant differences (*p* = .32 and *p* = .92 for BNP reduction and NYHA class improvement, respectively).

Among 11 patients milrinone therapy was interrupted; 3 for switching therapy to levosimendan (according to local policy and not induced by these patients' clinical condition), 1 patient underwent a left ventricular assist device (LVAD) implantation and 4 patients deceased (1 died out of hospital and the family refused autopsy, 1 suffered an intractable ventricular fibrillation [not during milrinone therapy] and 2 died because of sepsis). For social reasons, three patients were transferred to home care therapy.

## 4 DISCUSSION

In this small cohort of advanced HF patients, we were able to demonstrate the safety and the potential efficacy of repetitive intermittent IV milrinone therapy in stabilizing advanced HF patients and allowing them to better tolerate neurohormonal therapy. Our findings are reinforced by the fact that compared with recent HF trials,^7^, ^8^, ^9^ our small registry comprised advanced HF patients and included elderly, ischemic patients with high prevalence of significant kidney disease.

Hemodynamic studies demonstrated the potential beneficial effect of inotropes in HF. Specifically, cyclic AMP‐based inotropes (e.g., milrinone), were found to decrease systemic and pulmonary vascular resistance, improve cardiac output, and induce a smaller increase in oxygen consumption (compared with dobutamine or epinephrine), making them compelling agents for HF management.^10^, ^11^, ^12^, ^13^ Unfortunately, large‐scale clinical trials showed that despite these hemodynamic effects, long‐term inotropic therapy was associated with increased mortality rates.^14^, ^15^ Consequently, guideline documents advocate against the use of inotropes in most clinical scenarios and recommend to consider inotropic support either as a short‐term tool for clinical stabilization or as a mean to improve quality of life in end‐stage HF patients.^1^, ^16^ Nevertheless, contemporary small‐scale (usually single‐center) registries have reported better than expected clinical results in advanced HF patients receiving continuous inotropic support.^2^, ^17^ These findings might be explained by the prevalent use of beta‐blocking agents and ICDs in current practice. However, certain drawbacks in these trials are the use of cumbersome continuous IV pumps and the remaining high rates of mortality, LVAD implantations, heart transplantations, and hospitalizations in these patients.

We have chosen to use intermittent, repetitive, short‐term milrinone infusion in the setting of advanced HF outpatient unit operated by trained personnel. All patients had an ICD and were treated with beta‐blockers. As discussed above, the use of milrinone as the agent of choice was based on prior studies showing improved hemodynamic effect. However, we chose to test the option of intermittent infusion rather than continuous IV drip based on studies demonstrating that despite a half‐life of 4 h, intermittent milrinone had a favorable effect in advanced HF patients. Hatzizacharias et al.^18^ demonstrated that in a small cohort of advanced HF, four cycles of 48−72 h of milrinone given every 3 weeks had a statistically significant improvement on cardiac output, systemic and pulmonary vascular resistance lasting not only between cycles but also 4 months after the last cycle of milrinone infusion. Of note, the researchers in this trial chose to withheld angiotensin‐converting enzyme inhibitors therapy due to concern of profound blood pressure reduction after milrinone exposure. In another study, Marius Nunez et al.^19^ showed that advanced HF patients treated with intermittent 48‐h milrinone drip had lower rates of hospital admissions and emergency department visits. These data could be explained by a protracted effect of milrinone on intracellular calcium homeostasis and energy metabolism and also by possible mitochondrial structural changes.^20^, ^21^ Furthermore, these findings support the results of our observational study demonstrating that in advanced HF, stabilization can be achieved with the use of intermittent milrinone therapy. During milrinone therapy, we observed a decrease in BNP levels, despite the significant increase in the number of patients treated with ARNI (known to increase BNP levels) as well as a significant improvement in symptoms and functional class, which might be explained directly by milrinone therapy or indirectly by the clinical and hemodynamic stabilization achieved by milrinone utilization which allowed us to increase GDMT use. While the introduction to SGLT2 inhibitors might have contributed to the improvement in NYHA class (and though we cannot ascertain that milrinone therapy allowed our patients to better tolerate this therapy) we believe that the exposure to milrinone had also contributed to patients' stability because the improvement in NYHA class was observed also among patients not treated with SGLT2 inhibitors. A small increase in creatinine levels was observed, which could be explained by the increased use of SGLT2 inhibitors. Furthermore, we did not observe any deterioration in echocardiography parameters, including LVEF, left ventricular end systolic diameter, and left ventricular end diastolic diameter. A trend toward improvement in SPAP and E/e' was observed. Notably, opposed to older studies, we did not observe an excess risk of life‐threatening arrhythmias during IV milrinone therapy, a finding which might be explained by the prevalent use of beta‐blocker.^22^

Our study has several limitations; it is a small, observational unblinded study, conducted in a single center. Nevertheless, it represents advanced HF patients who were managed as outpatients and were free of continuous IV pump, a fact which probably contributed to improved quality of life. Also, a significant number of our patients were also treated with IV furosemide and iron, interventions that might have influenced their symptoms. Nevertheless, furosemide doses were either maintained or reduced along the follow‐up, suggesting that other contributing factors (e.g., milrinone and GDMT) could have improved the patients' clinical condition.

In conclusion, we present for the first time, to our knowledge, the use of repetitive, intermittent short‐term IV milrinone therapy as a safe and potentially efficacious treatment in ambulatory advanced HF patients. Future large‐scale randomized trials are needed to evaluate these initial results.

## CONFLICT OF INTERESTS

The authors declare no conflict of interests.

## Data Availability

The data that support the findings of this study are available from the corresponding author upon reasonable request.
